# Coping with unpredictable environments: fine-tune foraging microhabitat use in relation to prey availability in an alpine species

**DOI:** 10.1007/s00442-024-05530-1

**Published:** 2024-04-09

**Authors:** Davide Scridel, Matteo Anderle, Federico Capelli, Alessandro Forti, Chiara Bettega, Corrado Alessandrini, Maria del Mar Delgado, Luca Pedrotti, Piergiovanni Partel, Giuseppe Bogliani, Paolo Pedrini, Mattia Brambilla

**Affiliations:** 1 Department of Life Sciences, University of Trieste, via L. Giorgieri 10, 34127 Trieste, Italy; 2 Museo delle Scienze di Trento (MUSE), Ufficio Ricerca e Collezioni, Corso del Lavoro e della Scienza 3, 38122 Trento, Italy; 3 Institute for Alpine Environment, Eurac Research, viale Druso 1, 39100 Drususallee Bolzano/Bozen, Italy; 4 Department of Environmental Science and Policy, Milan University, via Celoria 26, 20123 Milan, Italy; 5 Department of Ecology, University of Innsbruck, Sternwartestrasse 15/Technikerstrasse 25, 6020 Innsbruck, Austria; 6 Biodiversity Research Institute (IMIB, CSIC-Oviedo University, Principality of Asturias), Campus Mieres, Mieres (Asturias), Spain; 7 Stelvio National Park, via de Simoni 42, 23032 Bormio, Italy; 8 Ente Parco Naturale Paneveggio-Pale di San Martino, località Castelpietra 2, 38054 Primiero San Martino di Castrozza, Trento Italy; 9 Department of Earth and Environmental Sciences, University of Pavia, via Ferrata 1, 27100 Pavia, Italy; 10 CRC Ge.S.Di.Mont, Milan University, sede di Edolo, via Morino 8, 25048 Edolo, BS Italy

**Keywords:** Mountain birds, Arthropods, Climate change, Foraging site fidelity, *Montifringilla nivalis*

## Abstract

Microhabitat utilisation holds a pivotal role in shaping a species’ ecological dynamics and stands as a crucial concern for effective conservation strategies. Despite its critical importance, microhabitat use has frequently been addressed as static, centering on microhabitat preference. Yet, a dynamic microhabitat use that allows individuals to adjust to fine-scale spatio-temporal prey fluctuations, becomes imperative for species thriving in challenging environments. High-elevation ecosystems, marked by brief growing seasons and distinct abiotic processes like snowmelt, winds, and solar radiation, feature an ephemeral distribution of key resources. To better understand species’ strategies in coping with these rapidly changing environments, we delved into the foraging behaviour of the white-winged snowfinch *Montifringilla nivalis*, an emblematic high-elevation passerine. Through studying microhabitat preferences during breeding while assessing invertebrate prey availability, we unveiled a highly flexible microhabitat use process. Notably, snowfinches exhibited specific microhabitat preferences, favoring grass and melting snow margins, while also responding to local invertebrate availability. This behaviour was particularly evident in snow-associated microhabitats and less pronounced amid tall grass. Moreover, our investigation underscored snowfinches’ fidelity to foraging sites, with over half located within 10 m of previous spots. This consistent use prevailed in snow-associated microhabitats and high-prey-density zones. These findings provide the first evidence of dynamic microhabitat use in high-elevation ecosystems and offer further insights into the crucial role of microhabitats for climate-sensitive species. They call for multi-faceted conservation strategies that go beyond identifying and protecting optimal thermal buffering areas in the face of global warming to also encompass locations hosting high invertebrate densities.

**Supplementary Information:**

The online version contains supplementary material available at 10.1007/s00442-024-05530-1.

## Introduction

Central to the study of animal ecology is the understanding of the processes by which animals select resources, by linking patterns of animal behaviour to underlying resource availability (e.g., Johnson 1980; Pyke 1984). This implies investigating what factors drive the use of certain habitats instead of others, according to a process generally referred to as habitat selection, which should allow a species to choose those habitats that better meet its ecological needs (Krebs and Davis 2012). Habitat selection has been investigated over very different functional, spatial and temporal scales (McGarigal et al. 2016).

A crucial aspect of habitat selection is the utilisation of microhabitats, which refers to spatio-temporally discrete areas of varying spatial scales (in ornithological literature between 1 and 707 m^2^; Morales et al. 2008; Patthey et al. 2012; see Alessandrini et al. 2022) that provide essential resources for various life-history functions (e.g., 4th order of habitat selection *sensu* Johnson 1980; Barbosa et al. 2010). Indeed, the use of the appropriate microhabitat influences individual fitness (Wagner and Fortin 2013; Bonner and Fritz 2015), both directly, by affecting species’ survival and reproductive success (Wilson 1998; Jedlikowski and Brambilla 2017), and indirectly by improving foraging efficiency (Biscardi et al. 2007; Bowler et al. 2019), reducing costs of thermoregulation (du Plessis et al. 2012) and providing shelter from predators (Skelhorn and Ruxton 2013). Furthermore, studies investigating microhabitat use are becoming increasingly important in the context of climate change, as small-scale habitats can maximise the resilience and resistance of populations (i.e., *refugia* sites; Barbosa et al. 2010; Suggit et al. 2011, Frey et al. 2016; Betts et al. 2017; Alessandrini et al. 2022). This may be pivotal in terms of biodiversity conservation, for example when the preservation of a species’ entire geographic range is unfeasible, but the conservation of its habitats and microhabitats may be achievable.

Foraging microhabitat selection has been largely investigated as a function of habitat characteristics and availability (Tsiakiris et al. 2009; Belotti et al. 2013; Eierman et al. 2014; Spear et al. 2020; Barras et al. 2020; Korniluk et al. 2021). Although it is generally assumed that a forager distribution occurs at the highest prey densities to maximise their intake rate (Holling 1959; Stephens and Krebs 1986; Zwarts and Wanink 1993; Wallace et al. 2015; Roder et al. 2020), there is evidence showing that the overlap between species occurrences and the distribution of key resources may be imperfect due to different constraints including habitat complexity (i.e., detectability; Martinez et al. 2010; Müller et al. 2012; Benoit-Bird et al. 2013; Liu et al. 2019), presence of predators (e.g., Brown 1988; Heithaus et al. 2002; Clare et al. 2023), density-dependent effects (Piersma 2012; DeRoy et al. 2020), as well as limitation associated with the sampling methodology (e.g., Hunsicker et al. 2011; Kuhn et al. 2015). In this regard, when both prey and microhabitat availability have been assessed, they have been often evaluated separately over different spatial or temporal extents (Guillemette et al. 1992; Johnson and Sherry 2001; Barbaro et al. 2008; Moreno-Rueda et al. 2018; Scridel et al. 2022), leading to a potential mismatch between the observation of predators and environmental data such as prey-predator distribution (Hunsicker et al. 2011; Kuhn et al. 2015). Modelling microhabitat use and prey availability measured at the same spatio-temporal scale is still rare in the literature (but see Blendinger et al. 2012; Müller et al. 2012; Schwemmer et al. 2016; Aung et al. 2022), even if this could lead to a more precise inference of whether a species is able to adjust its microhabitat use according to the presence of its prey items.

Fine-tuning foraging microhabitat selection is likely to be crucial for predators that depend on ephemeral food resources in unpredictable environments, such as aquatic ones, deserts, and high-elevation/latitude systems. These environments often exhibit a patchy distribution of ephemeral prey, which vary significantly in space and time (Boyd et al. 2016; Pavey 2021; Beerens et al. 2011, 2015a, b). So far, little attention has been given to species residing in high-mountain alpine systems, despite their exposure to spatially and temporally dynamic trophic changes. In these habitats, where local primary production is generally low, the input of wind-borne arthropod fallout plays a fundamental yet unpredictable role as a resource for various animals, including birds that directly prey on invertebrates found on snow patches (Antor 1994, 1995; Brambilla et al. 2017, 2018a, 2019). In addition, for many insectivorous species snow-melting margins and alpine grasslands are fundamental microhabitats to capture key prey (i.e., Coleoptera, Diptera, Lepidoptera), but their temporal availability, accessibility, and distribution may change on a daily basis depending on the rate of snowmelt (Muscio et al. 2005; Gobbi et al. 2006; Hågvar 2010; Brambilla et al. 2019; Resano-Mayor et al. 2019). In this regard, alpine birds can serve as ideal models for studying how animals adjust their foraging strategies to unpredictable and variable food resources in dynamic environments.

In this study, we explicitly evaluate the potential adjustment of microhabitat use according to fluctuating prey availability during the crucial phase of the nestling rearing period in the white-winged snowfinch *Montifringilla nivalis* (hereafter snowfinch). Our specific aims are: (i) to assess the relative variation in invertebrate availability across typical alpine microhabitats, and its relation to abiotic factors (e.g., temperature, wind and weather); (ii) to test whether snowfinches may tune the use of different microhabitats according to the site- and temporal-specific availability of potential prey among different types of microhabitats, and (iii) to determine if snowfinch pairs display a repeatable foraging behaviour by re-visiting the same profitable location and microhabitats after a successful foraging event (foraging site fidelity; e.g., Wakefield et al. 2015). We expect grassland and associated microhabitats to harbour the highest abundances and diversity of invertebrates, as snowfinches and other alpine birds have been reported to favour these microhabitats (Barras et al. 2020; Brambilla et al. 2017). Being ectotherms, we predict that invertebrates will be more abundant when temperatures are generally warmer. Given that breeding phenology in arctic and alpine species is tightly linked to the onset of spring/early summer snowmelt and associated growth of alpine vegetation, which causes a rapid increase in invertebrate availability on-site (Brambilla et al. 2018a; Saalfeld et al. 2019; Schano et al. 2021; Niffenegger et al. 2023), we predict that snowfinches will exhibit the ability to adjust their foraging preferences in response to the abundance of prey available at their location. In line with the principles of the optimal foraging theory (Schoener 1971), we further expect snowfinches to exhibit a repeated visitation pattern to profitable locations or microhabitats to maximise their access to available prey.

## Material and methods

### Study system

The snowfinch is a high-elevation Passeridae species breeding across the Palearctic mountains, from the Iberian region to the Tibetan plateau (Summers-Smith and Bonan 2020). In Europe, the subspecies *M. n. nivalis* is confined all-year-round to alpine, sub-nival, and nival habitats across the Cantabrian, Pyrenees, Corsica, Alps, Apennines, and Balkans Mountain ranges (Brambilla et al. 2020). During the nestling–rearing period, the adults feed their youngsters with a variety of arthropods (Cramp and Perrins 1994; Glutz von Blotzheim and Bauer 1997; Resano-Mayor et al. 2019) that are collected from alpine grasslands, snow patches, and snow-melting margins, and delivered at nests located in either natural or artificial cavities such as rock crevices, ski-pylons, crags in building and nest boxes.

**Fig. 1:**
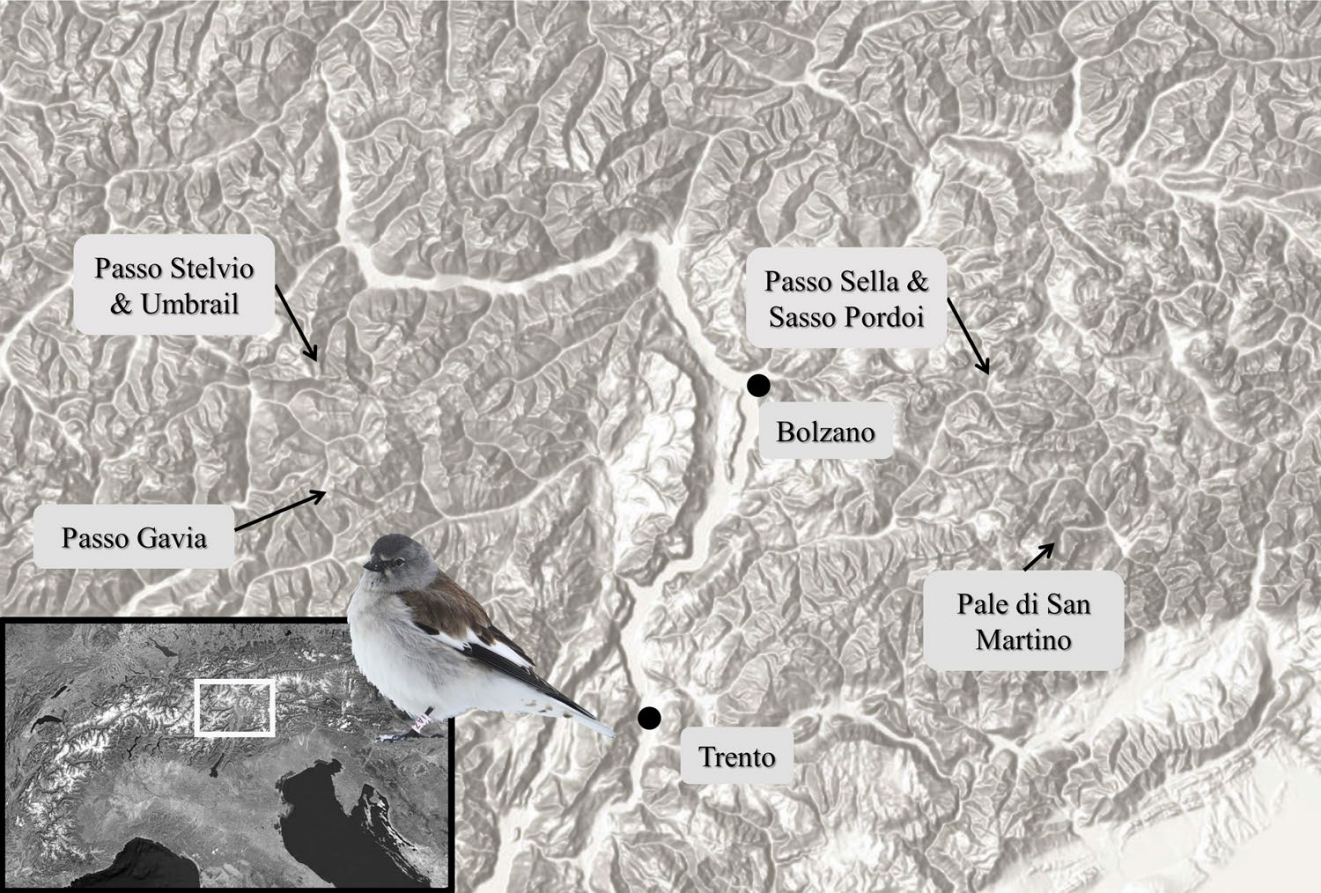
The bottom-left inset shows the location of the study area in the European Alpine Region while the main map, based on an hillshaded Digital Terrain Model (ESRI 2023), displays the locations of  the five main localities where the 15 breeding pairs of snowfinches were studied in the Central and Eastern Italian Alps (Italy). These localities include Passo Stelvio and Umbrail, Passo Sella, Sasso Pordoi, Passo Gavia, and Pale di San Martino. In addition, the location of Bolzano and Trento is also displayed on the map (black dots) to provide general orientation. Snowfinch photo credits: C. Bettega

Fifteen pairs of breeding snowfinches were studied in six main areas located between 2200 and 3020 m a.s.l. around mountain passes (Passo Sella *n* = 2, Sasso Pordoi *n* = 2, Passo Stelvio and Umbrail *n* = 9, Passo Gavia *n* = 1) and on a nival plateaux (Pale di San Martino *n* = 1) in the Central and Eastern Italian Alps (provinces of Sondrio, Brescia, Trento and Bolzano) (Fig. 1). These locations encompassed typical breeding snowfinch habitats above the treeline (i.e., alpine grassland, sub-nival and nival zones), from relatively unaltered alpine grasslands, and rocky plateaux to ‘anthropised’ mountain environments.

### Foraging microhabitat use and habitat characterisation

**Fig. 2:**
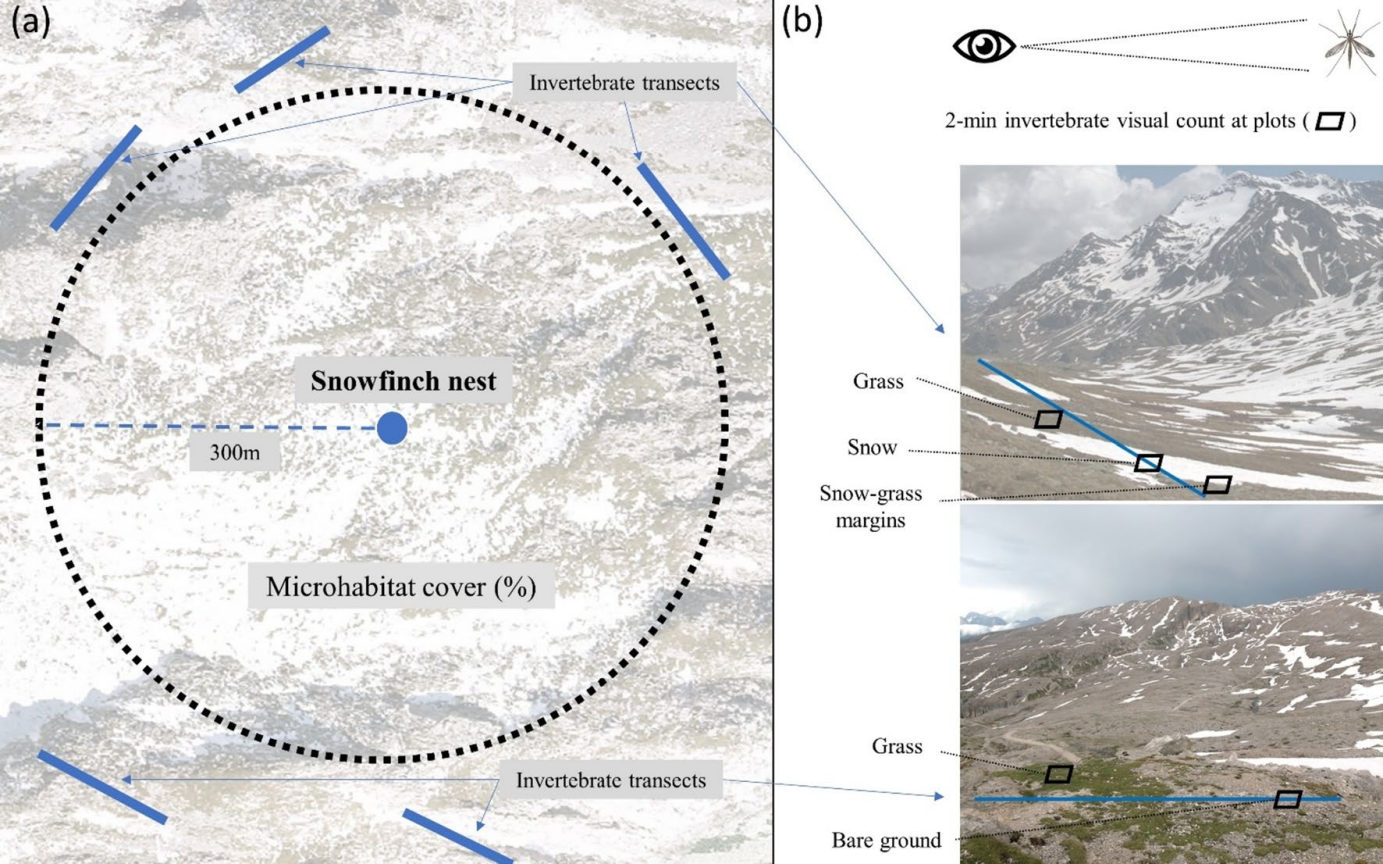
Graphical representation of the microhabitat and invertebrate sampling design used in this study. **a** To evaluate microhabitat availability surrounding the nest, estimates of habitat cover (%) were determined within a 300 m buffer (dashed black line) from the nest (blue point). To assess invertebrate availability, to avoid any effects associated with the food depletion by snowfinches, five transects (represented by blue lines) were identified in the field just outside the main foraging area of the snowfinches (300 m). These transects were chosen to encompass as many microhabitats as possible. **b** At each identified transect, 2-min visual counts of invertebrates were performed within 1 × 1 m plots for each available microhabitat in proximity of each transect (Photo credits: D. Scridel, Autonomous Province of Trento, Ortofoto Digitale 2019)

From the beginning of June to the end of July 2017, we conducted a comprehensive study by closely observing 15 breeding pairs of snowfinches for a full day (one pair/day), tracking their activities from their foraging microhabitat to the nest. For 60% of pairs (nine out of 15) we were also able to perform a second round of visits (hereafter “foraging sessions”), 3–4 days after the first one. Observations occurred mostly around midday (*x̄* = 1:30 p.m., range 8:00 a.m.–4:00 p.m.; UTC) to maximise the number of foraging events during the warmest hours of the day, when invertebrates are most active. As in other studies on snowfinch (e.g., Brambilla et al. 2017, 2019), we defined a foraging event as the last location of an individual with a beakful prior to a direct flight returning to the nest to feed its chicks. Our aim was to record approximately 20 consecutive foraging events per pair to ensure a robust and representative sample size. However, due to the complexity in working in such unpredictable environments (adverse weather and terrain), we were not always able to achieve this target. Indeed, the overall mean number of daily foraging events per pair was 17.9 SE ± 0.9, ultimately leading to a total of 411 foraging events recorded over 24 foraging sessions. We recorded the exact position (latitude and longitude) of each foraging location by means of a portable GPS and classified the microhabitat in 1m^2^ plots according to pre-defined types representing the most important foraging habitats for the species based on previous studies (see e.g., Brambilla et al. 2017, 2018a, 2018b, Resano‑Mayor et al. 2019). These were: (i) snow patch (snow cover ≥ 90%; hereafter “snow”); (ii) grassland (grass cover ≥ 90%, hereafter “grass”); (iii) margin snow patch-grassland (when not falling in the previous two categories; hereafter “snow-grass”); (iv) bare substrate (cover of bare ground, sand, rock, scree and/or boulders ≥ 90%; hereafter “bare”); (v) margin snow patch-bare substrate (hereafter “snow-bare”). We also assessed the relative availability of all five microhabitats within the average foraging range from the nest by estimating their coverage (%) in the field, and reviewed using the most recent satellite images offered by Google Earth (V 7.1.2.2041). For this assessment, we used a buffer with a radius of 300 m, which corresponds to the typical distance snowfinches travel for food during chick rearing (Strinella et al. 2007; Grangé 2008; Brambilla et al. 2019; Fig. 2). Habitats availability was not an issue in our study locations as all habitats were represented at each site, yet the coverage of each varied within the 300-m radius (Table S1).

### Invertebrate sampling and indexes

To investigate the ability of snowfinches to track prey over space and time, we collected data on invertebrate availability in all microhabitats immediately after the completion of each foraging sessions, typically 10–30 min later. To ensure that our assessment of invertebrate availability was not influenced by snowfinch predation depleting the local invertebrate population, we chose to sample at a slightly greater distance from each nest (*x̄* = 423.1 SE ± 46.4 m), yet remaining close enough to address potential preferences in nest placement linked to higher-than-average invertebrate densities (Niffenegger et al. 2023). This distance exceeds the previously reported mean maximum foraging distance of 300 m as described in earlier studies (Strinella et al. 2007; Grangé 2008; Brambilla et al. 2019). By employing this sampling strategy, we aimed to minimise any potential interference due to snowfinch predation while assessing invertebrate availability on-site. To obtain a balanced sample of invertebrate measures across all microhabitats, for each nest and foraging session we randomly identified five transects that included a transition of as many foraging microhabitats type as possible within an area (Fig. 2). In this way, we were able to obtain a general picture of invertebrate availability across multiple locations surrounding the nest. Once transects were identified, we performed 2 min visual counts in 1 m^2^ plots of all invertebrates according to each microhabitat (Fig. 2). These were performed standing still in close proximity of each plot (*ca.* 30–100 cm) and in the case of grass microhabitats we also actively searched invertebrates among the short swards (usually < 10–15 cm height). For each transect, we recorded additional data including the survey time (in hours), wind strength (absent: ≤ 0.5 m/s; weak/moderate: > 0.5 m/s and ≤ 20 m/s; strong: > 20 m/s), air temperature (in degrees Celsius; obtained from local meteorological stations), and sky cover (clear: 0–10% cloud coverage; partially clouded: 10–50%; clouded: ≥ 50%). Ultimately, we relied on 119 transects and 467 sampling plots for invertebrates (consisting of 149 in grass, 121 in snow, 90 in snow-grassland, 62 in snow-bare, and 45 in bare).

All arthropods present (dead or alive) in a given plot were counted to determine their index of abundance. They were also identified at the Order level to assess taxonomic richness (index of taxonomic richness). In addition, their body size was estimated by measuring the length from head to abdomen using a ruler and expressing it in millimeters, which provided the index of invertebrate size. We chose to assess all invertebrates present in a given plot, rather than concentrating on specific Orders for two reasons. First, while the general diet of snowfinches is known and includes many invertebrate Orders and Families (Diptera, Lepidoptera, Hymenoptera, Hemiptera, Arachnida, Coleoptera, Orthoptera, Ophistophora, Dermaptera; Cramp and Perrins 1994; Glutz von Blotzheim and Bauer 1997; Resano-Mayor et al. 2019), to date we lack quantitative studies assessing the relative contribution of each Order to their diet. Second, we aimed to present general patterns of invertebrate indices across typical alpine microhabitats, where little research has been done, which may serve as a useful reference for future studies. In addition, using this non-destructive method we could approximate well the availability and catchability of prey as captured by the snowfinch, that performs a visual search of their prey and forages by picking conspicuous ground-dwelling invertebrates standing out particularly well on high-elevation substrates (Antor 1995; Brambilla et al. 2017, 2018a, b). Visual counts are generally considered a reliable method, particularly for estimating abundances of butterflies (e.g., Pollard and Yates 1993), grasshoppers (e.g., Wettstein and Schmid 1999), Hymenoptera (e.g., Gunnarsson and Federsel 2014) and spiders (e.g., Costello and Daane 1997). Methods such as pitfall traps, sweep nets, sticky boards, and suction samplers, despite being commonly used, may not capture invertebrates in a way that accurately reflects the snowfinch foraging behaviour because the latter hunts by sight in daylight conditions.

### Statistical analysis

To comprehensively assess variations in invertebrate availability among different microhabitat types, we developed models for three indices (abundance, richness, and size) that collectively represent the average values at the plot level (1 × 1 m). These indices were uncorrelated between each other (abundance vs richness *r* = 0.17, abundance vs size *r* = 0.03, size vs richness *r* = 0.01) and therefore treated as separate response variables and modelled using Linear Mixed Models (“LMM”; for invertebrate size index—body length in mm: Gaussian distributed and log-transformed) and Generalized Linear Mixed Models (“GLMM”; for invertebrate abundance and richness indexes–overdispersed count data: negative binomial). The models were fitted using the R package ‘*glmmTMB*’ (Brooks et al. 2017). The fixed effects included in all three models were the microhabitat type (categorical variable with 5 levels), the date of the survey (continuous variable, represented as days since January 1st), the time of the survey (continuous variable, restricted between 9 am and 5 pm, UTC), sky cover (categorical variable with 3 levels), temperature (continuous variable), and wind strength (categorical variable with 3 levels). Prior to model fitting, potential quadratic effects were explored for all continuous variables, but ultimately only the time variable (hours) and day of survey were retained in the models as statistically significant for their quadratic effect (*p* < 0.05). A random effect structure was needed to account for the non-independency of repeated measures of invertebrates sampled in the same mountain areas (i.e., Pordoi, Gavia, Stelvio, Sella, Rosetta), repeated visits at nests and multiple microhabitats within transects. We used Akaike’s information criterion corrected for small sample size (AICc; Burnham and Anderson 2002) to test the best performing (lowest AICc) random effect structure via Restricted Maximum Likelihood, which resulted in the inclusion of only the nest and transect (i.e., ‘(*1|Nest ID/Transect ID*)’), but not the mountain area.

We evaluated the snowfinches’ ability to selectively use different microhabitats based on the specific availability of prey in different locations and time periods. The dependent variable was created using the ‘*cbind*’ function to combine two vectors: one representing the number of foraging locations in a given microhabitat (e.g., grass), and the other representing the number of foraging locations in all the other microhabitats. The fixed effects were the reference type of microhabitat (categorical variable with 5 levels), the relative percentage cover of the microhabitat within a 300 m-radius buffer from each nest, the three indexes of invertebrate availability (i.e., abundance, richness and body size) averaged at microhabitat and nest level, and the date of the survey (continuous variable represented as days since January 1st). In that way, the model explored how the preferential use of a microhabitat over the others is affected by its type and availability, invertebrate traits, and seasonality. A categorical variable named “*Nest ID*”, identifying each nest, was included as random factor. To account for overdispersion, we worked with a betabinomial GLMM fitted via the *R* package ‘*glmmTMB*’ (Brooks et al. 2017). *R*^2^ values were calculated to estimate the variance explained by fixed factors only (marginal *R*^2^: R^2^m) or by both fixed factors and random factors together (conditional *R*^2^: R^2^c) according to Nakagawa and Schielzeth (2013).

Foraging site fidelity was evaluated at the nest level by calculating the Euclidean distance of consecutive foraging location within each foraging session and according to each microhabitat type using the package ‘*geosphere*’ (Hijmans 2021). To spatially assess whether the use of certain microhabitats was more repeatable than others, we fitted LMMs with the log-transformed variable of the distance as response variable, while fixed effects were the microhabitat type and the three measures of invertebrate availability. A foraging session was set as random intercept factor. The interaction between habitat and invertebrate indexes was tested a priori and found not to be significant (p > 0.05).

All analyses were performed in R software (version 4.0.4; R Core Team 2021). Models were tested for within-group collinearity by calculating the variance inflation factor (VIF) using the package ‘*car*’ (Fox and Weisberg 2019). Explanatory variables with VIF value > 4 were removed from the model and multicollinearity was re-checked to verify that the remaining variables were not correlated (Zuur et al. 2009). All predictors were scaled and centered at the mean for better interpretation of coefficient and to improve model convergence. We evaluated whether all our models met linearity assumptions (linearity, independence, normality of errors, equal variance), using the R packages ‘*dharma*’ (Hartig 2020) and ‘*performance*’ (Lüdecke et al. 2021).

## Results

### Invertebrate availability in high-elevation microhabitats

**Fig. 3:**
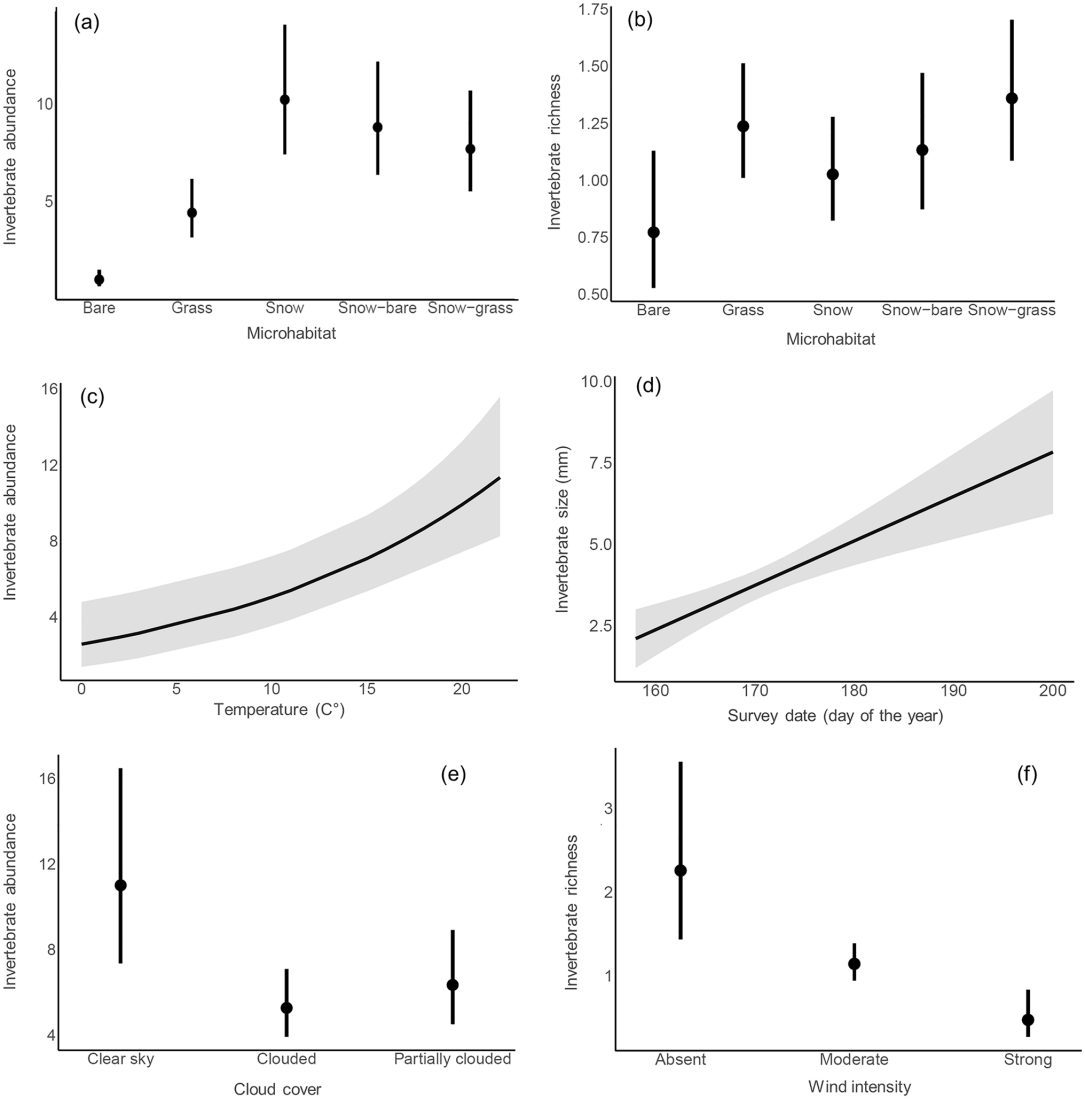
Some of the most important abiotic (temperature (**c)**, day of the survey (**d)**, cloud cover (**e)**, wind intensity (**f)**), and biotic predictors (microhabitat (**a**,**b)**) associated with the variation in invertebrate availability (abundance, size and richness) in high-elevation alpine systems according to the analyses presented in Table 1. Plots represent mean (dot) and ± 95% confidence intervals (whiskers, shaded area)

Overall, a total of 3,465 invertebrates were recorded during invertebrate visual counts. The mean number of invertebrates detected per microhabitat varied greatly, but were generally more abundant on snow-associated microhabitats (snow: *x̄* = 9.83, 95% CI = 6.69–13.87; snow-bare: *x̄* = 8.00, 95% CI = 5.50–11.65; snow-grass: *x̄* = 7.17, 95% CI = 4.99–10.30) and were the lowest on bare substrate (*x̄* = 1.74, 95% CI = 1.09–2.79; Table 1, Fig. 3). Patterns of invertebrate size followed a similar trend, being larger on snow-associated microhabitats (snow: *x̄* = 1.25, 95% CI = 1.10–1.39; snow-bare: *x̄* = 1.19, 95% CI = 1.02–1.37; snow-grass: *x̄* = 1.08, 95% CI = 0.92–1.34). Grass (*x̄* = 1.24, 95% CI = 1.01–1.51) and snow-grass margins (*x̄* = 1.36, 95% CI = 1.08–1.70) hosted the highest richness of invertebrate Orders. A quadratic relationship was detected for invertebrate abundances in relation to survey date (peaking towards the end of June) and to the time of the surveys (peaking between 1 and 2 p.m.; Table 1). On the other hand, invertebrate size and richness increased linearly as the seasons progressed (Fig. 3). Highest abundances, size and richness of invertebrates were detected during conditions of clear skies, absence of winds and warmer temperatures (Table 1, Fig. 3). Values of the marginal *R*^2^ were higher for the index of invertebrate abundance (0.68) compared to those of size (0.12) and richness (0.33).

**Table 1:** Linear (invertebrate size) and Generalized (invertebrate abundance and richness) Mixed Effect Models’ results describing predictors of invertebrate availability across five types of microhabitats (reference level: bare substrate), day of the survey, hour, sky cover (reference level: clouded), temperature, and wind strength (reference level: absent). Standardised estimates are shown alongside 95% confidence intervals, test statistics (Wald’s *χ*^2^ for invertebrate abundance and richness, F statistics for size), degrees of freedom (df), and *P* value (*P*)

Predictors	Estimate	std.error	*df*	*χ*^2^*/F*	*P*
Index of invertebrate abundance					
Microhabitat (grass)	**0.93 [0.54,1.32]**	**0.2**	**4**	**112.62**	**<0.0001**
Microhabitat (snow)	**1.72 [1.34,2.11]**	**0.19**	**4**		**<0.0001**
Microhabitat (snow-bare)	**1.52 [1.12,1.93]**	**0.21**	**4**		**<0.0001**
Microhabitat (snow-grass)	**1.4 [0.99,1.81]**	**0.21**	**4**		**<0.0001**
Survey date	**6.94 [2.24,11.64]**	**2.39**	**1**	**8.38**	**0.004**
Survey date^2^	**− 6.87 [− 11.48, − 2.26]**	**2.35**	**1**	**8.52**	**0.003**
Hour	**3.61 [1.52,5.69]**	**1.06**	**1**	**11.55**	**0.0007**
Hour^2^	**− 3.28 [− 5.28, − 1.28]**	**1.02**	**1**	**10.35**	**0.001**
Sky cover (clouded)	** − 0.74 [− 1.08, − 0.39]**	**0.18**	**2**	**17.63**	**<0.0001**
Sky cover (partially clouded)	** − 0.55 [− 0.92, − 0.17]**	**0.19**	**2**		**<0.0001**
Temperature	**0.43 [0.22,0.64]**	**0.11**	**1**	**16.06**	**<0.0001**
Wind (strong)	**− 0.89 [− 1.66, − 0.14]**	**0.39**	**2**	**7.02**	**0.019**
Wind (weak/moderate)	**− **0.26 [**− **0.96,0.44]	0.36	2		0.465
*Conditional R*^*2*^*: 0.91; marginal R*^*2*^*: 0.68*					
Index of invertebrate size					
Microhabitat (grass)	0.11 [**− **0.12,0.33]	0.11	4	**13.24**	0.346
Microhabitat (snow)	**0.31 [0.09,0.54]**	**0.11**	**4**		**0.006**
Microhabitat (snow-bare)	**0.26 [0.02,0.49]**	**0.12**	**4**		**0.035**
Microhabitat (snow-grass)	0.14 [**− **0.09,0.38]	0.12	4		0.222
Survey date	**0.25 [0.13,0.37]**	**0.06**	**1**	**16.8**	**<0.0001**
Hour	0.07 [**− **0.03,0.17]	0.05	1	1.75	0.185
Sky cover (clouded)	**− 0.36 [− 0.56, − 0.15]**	**0.1**	**1**	**12.19**	**<0.0001**
Sky cover (partially clouded)	**− **0.14 [**− **0.36,0.06]	0.11	1		0.185
Temperature	**− **0.01 [**− **0.13,0.11]	0.06	2	0.02	0.893
Wind (strong)	**0.46 [0.03, 0.90]**	**0.22**	**2**	**4.58**	**0.036**
Wind (weak/moderate)	0.17 [**− **0.18,0.52]	0.18	1		0.337
*Conditional R*^*2*^*: 0.19; marginal R*^*2*^*: 0.12*					
Index of invertebrate richness					
Microhabitat (grass)	**0.47 [0.08,0.86]**	**0.19**	**4**	**10.59**	**0.017**
Microhabitat (snow)	0.28 [**− **0.11,0.68]	0.2	4		0.156
Microhabitat (snow-bare)	0.38 [**− **0.02,0.79]	0.21	4		0.067
Microhabitat (snow-grass)	**0.57 [0.16,0.96]**	**0.2**	**4**		**0.005**
Survey date	**− **0.07 [**− **0.26,0.11]	0.09	1	0.58	0.447
Hour	0.09 [**− **0.03,0.23]	0.07	1	2.11	0.146
Sky cover (clouded)	**− 0.33 [− 0.59, − 0.06]**	**0.13**	**2**	**8.13**	**0.013**
Sky cover (partially clouded)	**− 0.35 [− 0.63, − 0.07]**	**0.14**	**2**		**0.014**
Temperature	**0.28 [0.12,0.44]**	**0.08**	**1**	**11.5**	**0.0006**
Wind (strong)	**− 1.56 [− 2.23, − 0.9]**	**0.34**	**2**	**21.49**	**<0.0001**
Wind (weak/moderate)	**− 0.68 [− 1.21, − 0.16]**	**0.27**	**2**		**0.012**
*Conditional R*^*2*^*: 0.39; Marginal R*^*2*^*: 0.33*					

Predictors with significant *P*-values (*α* = 0.05) and with 95% confidence intervals not overlapping zero are shown in bold. Conditional and marginal R^2^ are shown at the bottom of each section of the table.

### Fine-tune foraging microhabitat use in relation to microhabitat and prey availability

Out of the 411 foraging observations recorded, the most frequent microhabitat used by snowfinch was grass (mean number of observation/session : *x̄* ± SE = 7.2 ± 1.4, *n* = 151 or 37% of all events), followed by snow-grass, which accounted for 32% of all observations (*x̄* ± SE = 6.3 ± 1.4, *n* = 132), snow-bare substrate with 14% of all observations (*x̄* ± SE = 3.2 ± 0.6, *n* = 58), snow with 13% of all observations ( *x̄* ± SE = 2.4 ± 0.7, *n* = 55), and bare substrate with 4% of all observations ( *x̄* ± SE = 1.3 ± 0.2, *n* = 15).

**Fig. 4:**
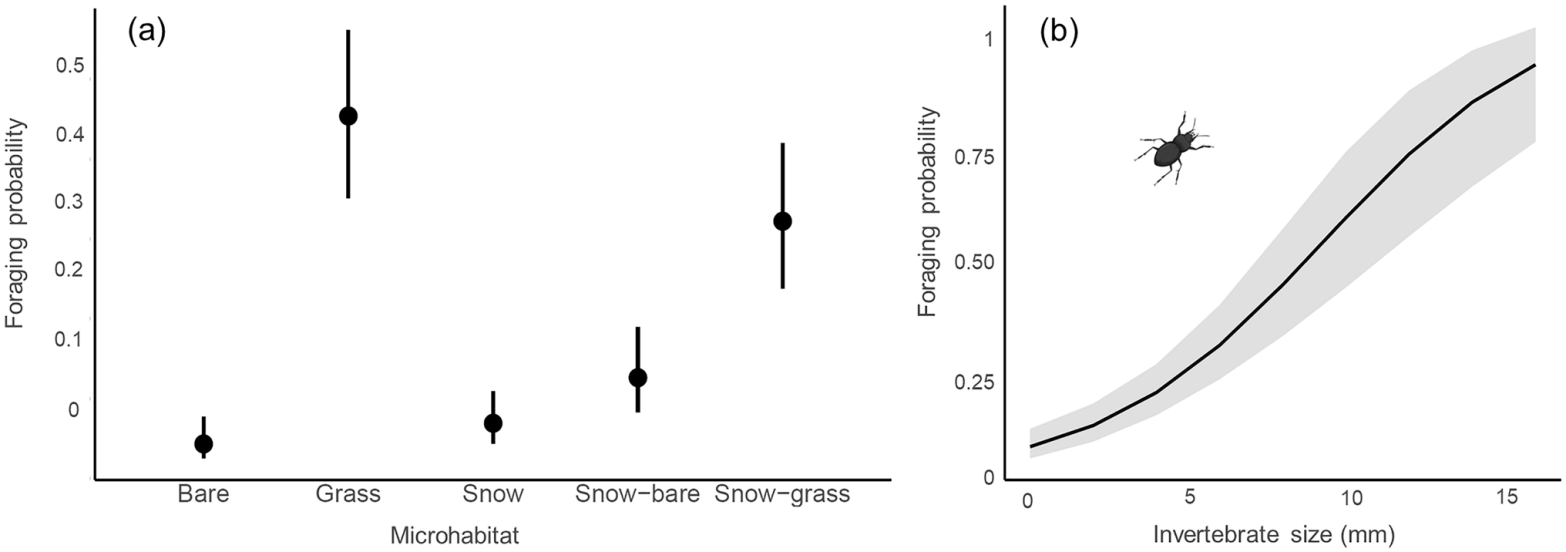
Snowfinch foraging probability during the breeding period in relationship to microhabitat type (**a)** and invertebrate size (**b)** according to the model presented in Table 2. Snowfinches fine-tuned their foraging behaviour based on microhabitat type and invertebrate size during the breeding period. Simultaneous field observations of foraging and invertebrate sampling demonstrate that snowfinches have a preference for specific microhabitats, primarily grass and snow-grass margins. In addition, they displayed the ability to adjust their foraging use based on areas associated with higher invertebrate abundance, showcasing their flexibility in resource selection

The importance of microhabitat type for snowfinch foraging behaviour was confirmed by our model, which indicated that snowfinches had a higher probability of foraging on grass and snow-grass compared to other microhabitats (see Table 2, Fig. 4). Notably, our analysis revealed a strong positive relationship between foraging probability and invertebrate size, while a slightly weaker but still positive relationship was observed for invertebrate abundance (*p* = 0.055).

**Table 2:** Generalized Linear Mixed Effect Model describing the probability of snowfinch microhabitat use for foraging in relation to microhabitat types (reference level: bare substrate) and availability (% cover of each microhabitat within 300 m from the nest), and prey (indices of invertebrate abundance, size, and richness)

*Predictors*	*Estimate*	*std.error*	*df*	*χ*^2^	*P*
Invertebrate abundance	0.32 [− 0.01,0.64]	0.17	1	3.66	0.055
Invertebrate size	**0.49 [0.18,0.81]**	**0.16**	**1**	**9.3**	**0.002**
Invertebrate richness	0.11 [− 0.23,0.45]	0.17	1	0.41	0.521
Microhabitat (grass)	**1.7 [0.60,2.77]**	**0.55**	**4**	15.33	**0.002**
Microhabitat (snow)	0.06 [− 1.19,1.30]	0.64	4		0.927
Microhabitat (snow-bare)	0.57 [− 0.69,1.82]	0.65	4		0.373
Microhabitat (snow-grass)	1.16 [− 0.09,2.41]	0.64	4		0.069
Habitat cover	− 0.06 [− 0.46,0.33]	0.20	1	0.17	0.745
Survey date	− 0.04 [− 0.39,0.30]	0.18	1	0.07	0.806
*Conditional R*^*2*^*: 0.30; Marginal R*^*2*^*: 0.29*					

Standardised estimates are shown alongside 95% confidence intervals, test statistics (Wald’s χ^2^), degrees of freedom (df) and *P* value (*P*). Predictors with significant *P*-values (*α* = 0.05) and with 95% confidence intervals not overlapping zero are shown in bold. Conditional and marginal *R*^2^ are shown at the bottom of the table

### Foraging site fidelity

**Fig. 5:**
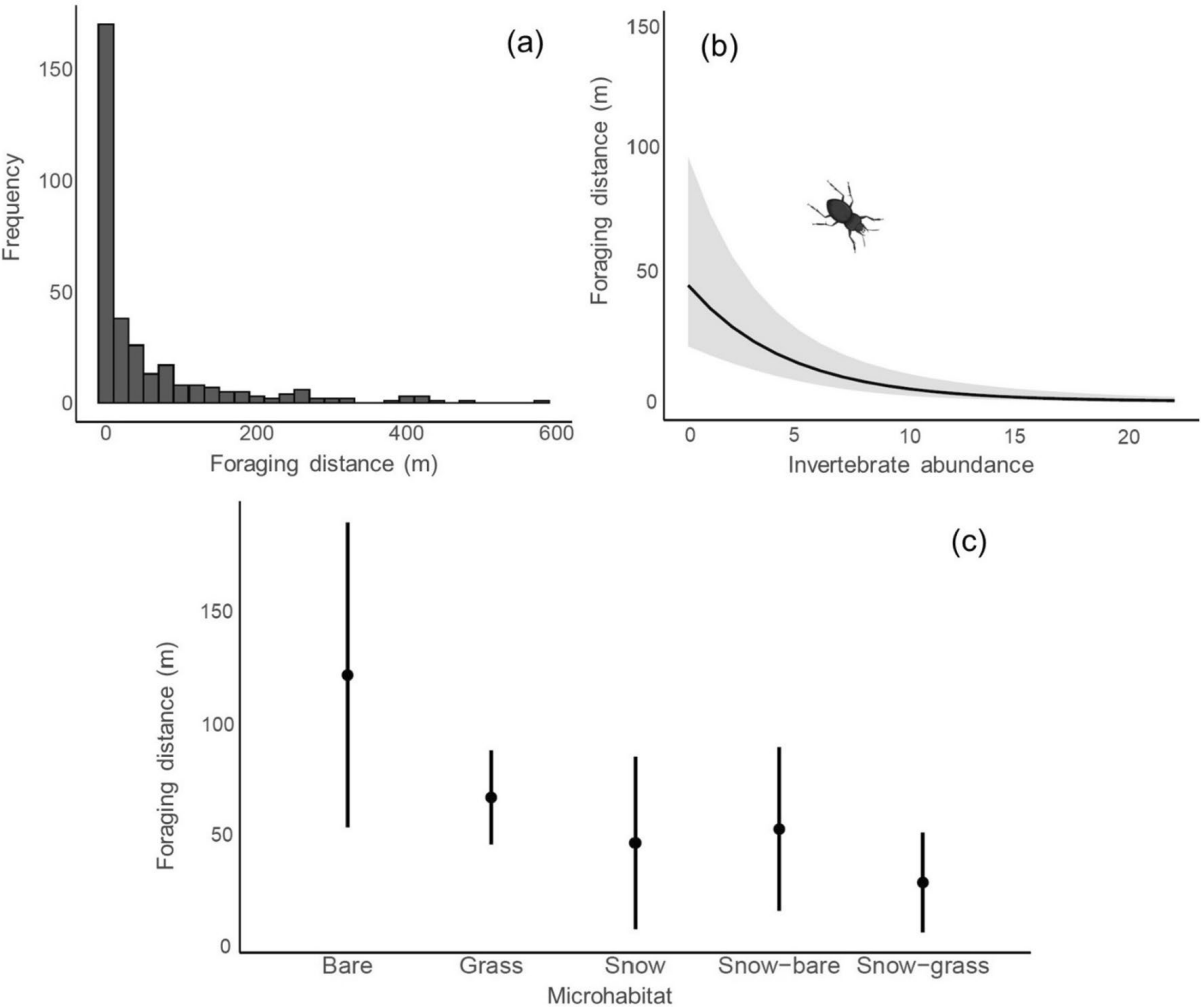
Relationship between distances of consecutive foraging locations according to microhabitat type and invertebrate availability as for the model presented in Table 3. (**a)** The frequency distribution of distances between successive foraging locations revealed a highly repetitive foraging behaviour among breeding pairs of snowfinches. These pairs tended to forage in extremely close proximity to previously visited locations, indicating a strong foraging site fidelity during the breeding period. (**b)** A negative relationship was observed between foraging distances and invertebrate abundance, indicating that snowfinches had a tendency to revisit locations with higher invertebrate densities. This suggests a flexible foraging strategy, where snowfinches actively seek out areas with abundant invertebrate prey. (**c)** Analysis of consecutive distances between foraging locations according to microhabitat type reveals that snowfinches exhibit variations in foraging location distribution. Specifically, foraging locations on bare and grass microhabitats tend to be more widely dispersed compared to locations associated with snow-associated microhabitats suggesting that the latter may offer more patchy and concentrated resources, leading to a closer clustering of foraging sites for snowfinches. Plots represent mean (dot) and ± 95% confidence intervals (whiskers, shaded area)

Overall, snowfinches displayed a high foraging site fidelity at pair level, with more than 50% (*n* = 211/411) of consecutive foragings being less than 10 m from the previous location (*x̄* = 56.88 m; range 0–361 m; Fig. 5). Mean consecutive foraging distances varied according to microhabitat. Birds that foraged on bare microhabitats tended to forage at greater distance compared to birds repeatedly foraging on grass and snow-associated microhabitats (Table 3; Fig. 5). We found a highly significant negative relationship (p < 0.0001) between the proximity of consecutive foraging sites at pair level and the mean invertebrate abundance, suggesting that snowfinches return to the same patch also when the mean invertebrate abundance is high, while the opposite trend was found in relationship to invertebrate richness.

**Table 3:** Linear Mixed Effect model describing the relationship between distances of consecutive foraging location (log-transformed response variable) and three predictors of invertebrate availability (abundance, size, and richness) and microhabitat (reference level: bare substrate). In bold are shown statistically significant invertebrate predictors according to *α* = 0.05 (reference level for microhabitat: bare substrate)

Predictors	Estimate	std.error	*df*	*F*	*P*
Invertebrate abundance	**− 1.20 [**− **1.74,-0.66]**	**0.91**	**1**	**20.40**	** < 0.0001**
Invertebrate size	− 0.29 [− 0.74,0.16]	0.27	1	1.26	0.265
Invertebrate richness	**0.77 [0.30,1.24]**	**0.23**	**1**	**10.62**	**0.001**
Microhabitat (grass)	− 1.66 [− 3.47,0.13]	0.24	4	4.73	0.070
Microhabitat (snow)	− 1.80 [− 3.82,0.21]	0.92	4		0.080
Microhabitat (snow-bare)	− 1.62 [− 3.45,0.20]	0.93	4		0.080
Microhabitat (snow-grass)	− **3.06 [**− **4.97,**− **1.14]**	**0.98**	**4**		**0.002**
*Conditional R*^*2*^*: 0.34; Marginal R*^*2*^*: 0.20*					

Standardised estimates are shown alongside 95% confidence intervals, test statistics (F), degrees of freedom (df) and *p* value (P). Predictors with significant *P*-values (*α* = 0.05) and with 95% confidence intervals not overlapping zero are shown in bold. Conditional and marginal *R*^2^ are shown at the bottom of the table

## Discussion

In challenging environments characterised by scarce and unevenly distributed resources, the ability of animals to adjust their use of microhabitats in response to dynamic changes in resource availability is likely crucial for their survival (Scheffers et al. 2014; Delaney et al. 2016). The white-winged snowfinch, a specialist of high-elevation habitats, in this context serves as an exemplary flagship species, which we refer to as endangered and charismatic species chosen to symbolise a specific habitat (i.e., high-elevation climate-sensitive habitats), which requires protection (Verissimo et al. 2011).

During the breeding period, snowfinches exhibit a well-established hierarchical pattern of microhabitat selection, with a preference for climate-sensitive microhabitats such as short-sward alpine grass and snow-grass margins (Brambilla et al. 2017; Alessandrini et al. 2022). Our research not only confirmed this general trend but also highlighted the snowfinch remarkable capacity to fine-tune the relative use of different microhabitats in response to changes in prey availability. Furthermore, our study provided novel insights into the foraging behaviour of a high-elevation species: instead of continually seeking new foraging locations, snowfinches exhibited a repetitive foraging behaviour, consistently capturing prey in specific sites, especially where invertebrates were abundant. This strong site fidelity in foraging behaviour might be a widespread trait among species inhabiting high-elevation environments characterised by rapidly changing resources. It allows species to conserve limited energy and optimise the fitness of both adult birds and their offspring by maximising prey capture.

### Invertebrate availability at high-elevation

Studies describing patterns of invertebrate availability for birds at high-elevation are limited in the literature and our work bridged some important knowledge gaps in these regards (Antor 1994, 1995; Hågvar 2010; Besimo 2019; Barras et al. 2022). As expected for ectothermic fauna, the abundance and diversity of invertebrates were strongly influenced by abiotic factors. Measures of arthropod abundance and richness were positively correlated with higher temperatures, either during the season or throughout the day. Likewise, the absence of cloud cover (i.e., clear skies with sunny conditions) was linked to increased availability of arthropods. Conversely, the presence of strong wind conditions resulted in reduced numbers of invertebrates, possibly due to wind gusts surpassing the flight capabilities of insects. When accounting for these factors, our models still revealed a substantial variability of invertebrates among microhabitats, providing further evidence for the complex distribution of arthropods in high-elevation environments (Seeber et al. 2022). The rapid changes in surrounding habitats caused by the short seasonality and variable rates of snowmelt are likely to play a crucial role in shaping the dynamic nature of the prey distribution in these environments (de Zwaan et al. 2023). Higher abundance of invertebrates was detected on snow-associated microhabitats, less on grass and the lowest on bare microhabitat, matching results of previous studies (Besimo 2019; Resano-Mayor et al. 2019). While it is generally assumed that large areas of snow cover may pose limits to primary productivity (i.e., vegetation growth followed by arthropods emergence), invertebrate fallout found directly on snow patches can be easily detectable and accessible by many mountain birds (Antor 1995). Indeed, in our study areas, fallout was composed by a great variety of invertebrate Orders such as bugs (Hemiptera: Aphididae; including various species of forest aphids *Cinara spp.*), beetles (Coleoptera; including some specimens of Colorado potato beetle *Leptinotarsa decemlineata*), flies (Diptera), midges (Diptera: Culicoides), ants (Hymenoptera). The literature has long described the dynamic pattern of wind-blown invertebrate influxes from lowlands, which can be observed on mountain tops worldwide (Mani 1968; Antor 1994; Rosvold 2016). We hypothesise that the greater abundance of invertebrates in snow, as compared to grass microhabitats, is likely due to the continuous accumulation of arthropods blown by the wind over several days and by the presence of larvae commonly found in the near snow front (Besimo et al. 2019). In contrast, invertebrates in grass microhabitats, such as adult beetles and crickets, move more quickly and do not accumulate in the same manner. Moist microhabitats created by melting snow (i.e., margin snow- grass and snow-bare) were also particularly rich in invertebrates. They are renowned to be particularly suitable for pupating craneflies (Diptera: Tupilidae) and Lepidoptera, the former being a keystone invertebrate prey for many mountain and upland birds across various latitudes, including the snowfinch (Tryon and MacLean 1980; Galbraith et al. 1993; Pearce-Higgins and Yalden 2004; Resano-Mayor et al. 2019).

### Dynamic microhabitat use: tuning responses to changing conditions

Foraging microhabitat use by breeding snowfinches has been investigated multiple times, especially in the Alps (e.g., Brambilla et al. 2017; Resano-Mayor et al. 2019, Alessandrini et al. 2022). Our results are in line with the previously described preferences for grass, snow margins and snow patches, while few foraging events occur on bare substrate (Antor 1995; Strinella et al. 2007; Brambilla et al. 2017, 2018a, b; Bettega et al. 2020, Alessandrini et al. 2022). Our results, together with available studies on this and other mountain species, suggest that the use for these microhabitats is influenced by both the availability and accessibility of prey, resulting in a preference for low-sward grass and for the interface between grass and snow (Resano-Mayor et al. 2019; Barras et al. 2020, 2022).

Species living in harsh and dynamic environments (i.e., where resources change at a relatively fast rate) may be expected to be able to track resources along their temporal and spatial variation. In these instances, the presence of species may not necessarily correlate with static measures of habitat quality (Kunegel-Lion et al. 2022). To our knowledge, such behavioural adjustment has been only limitedly demonstrated in animals in general (i.e., bats; Müller et al. 2012), with the few avian examples investigating waders feeding in accordance to changing water levels and prey availability (Beerens et al. 2011; 2015a, b; Schwemmer et al. 2016; Aung et al. 2022). Snowfinches, and likely other high-elevation mountain birds, must also adjust to resources that change quickly in relation to changes in snow cover and to unpredictable influxes of invertebrate fallout during spring and summer. In our study, snowfinches showed a strong response to invertebrate abundance and size, offering valuable insights for the conservation of climate-sensitive species. Indeed, these findings suggest that protective measures should not only target cold microhabitats providing optimal thermal buffering, such as small nival valleys where snow patches persist (sensu Brambilla et al. 2018b; Alessandri et al. 2022), but also encompass areas with high densities of invertebrates where these species tend to occur or accumulate as fallout.

### Repeatable foraging behaviour

Our analysis on foraging site fidelity revealed fascinating insights into the foraging behaviour of snowfinches. Notably, we observed that these birds possess the remarkable ability to spatio-temporally track their resources. They seem to maximise their energetic budget by repeatedly re-visiting previously foraged patches, rather than constantly exploiting new sites. Impressively, approximately 50% of the 411 foraging observations occurred within a mere 10 m from the previous location. The strong correlation we found between the proximity of consecutive foraging areas and the abundance of invertebrates strongly suggests that snowfinches consistently follow a pattern of re-visiting highly productive prey sites. This strategy allows them to optimise prey capture while conserving precious energy, making it a highly efficient foraging technique. Our findings align with the existing research on other species, where commuting to the same area has been linked to high prey capture rates (e.g., Thums et al. 2011; Carroll et al. 2018). This outcome not only supports our initial hypothesis but also aligns with the general principles of optimal foraging theory (Schoener 1971). Such a foraging strategy was indeed anticipated for species residing in challenging environments characterised by resource scarcity and the elevated physiological demands associated with life at high altitudes. This analysis, the first to our knowledge to be performed on high-elevation species, revealed variation in repeatability also according to microhabitat type, with snow-associated and grass microhabitats being re-visited more frequently than bare substrate. Such results might be explained by predation efficiency, which was probably higher in specific snow-grass edges, where invertebrates may be more detectable and abundant (Besimo 2019; Resano-Mayor et al. 2019). Although snowfinches displayed a higher frequency of selection for grass microhabitats during foraging, the presence of highly mobile invertebrates could lead snowfinches to track prey that are concealed amidst the grass across different locations. In contrast, invertebrates pupating on melting margins or those falling directly onto snow patches may be more “sessile”, resulting in a higher level of foraging site fidelity observed in these microhabitats. It is possible that the movement and availability of prey within grass microhabitats pose challenges for snowfinches. Further investigations incorporating measurements of sward height and detailed assessments of prey distribution and mobility within different microhabitats would provide a more comprehensive understanding of the factors influencing microhabitat foraging behaviour in snowfinches and similar species in high-elevation environments.

### Conclusion

Although mountain regions and species inhabiting them are generally poorly studied (Scridel 2014; Scridel et al. 2018; de Zwaan et al. 2022; Alba et al. 2022), they are valuable and novel model systems to investigate how species have evolved to live in extreme and unpredictable environments. This includes evaluating the plasticity of species’ foraging behaviour and the associated capacity in performing dynamic microhabitat use, which should allow them to modulate the use of microhabitats on the basis of the stochastic prey availability typical of these environments. The strong foraging site fidelity shown by snowfinches might have several important implications for conservation strategies. For example, an important question that arises is whether snowfinches show a long-term (multi-annual) tradition to re-visiting the same foraging location. If this was true, conservation actions target at the preserving very specific sites also at very fine scales is fundamental.

Both mechanisms of dynamic microhabitat use and foraging site fidelity can be solidly interpreted as adaptations to the demanding conditions in high-elevation mountain systems. Lastly, disentangling the dynamism of microhabitat use could also improve our understanding of ultimate (dynamic) drivers of species-environment relationships, thus adding further solid bases to develop effective conservation and management strategies, especially in the optic of climate change.

### Supplementary Information

Below is the link to the electronic supplementary material.

Supplementary file1 (DOCX 34 KB)

## Data Availability

The data that support the findings of this study are available from the corresponding author, [DS], upon reasonable request.
