# Supplementary material for: Coping with unpredictable environments: fine-tune foraging microhabitat use in relation to prey availability in an alpine species
Source: Oecologia. 2024 Apr 9;204(4):845–60. doi: 10.1007/s00442-024-05530-1 (PMC11062978; doi:10.1007/s00442-024-05530-1)
Supplement: Supplementary file 1 — Supplementary file1 (DOCX 34 KB) [file 442_2024_5530_MOESM1_ESM.docx]

*SUPPLEMENTARY MATERIALS*

**Coping with unpredictable environments: fine-tune foraging microhabitat use in relation to prey availability in an alpine species**

Davide Scridel^a,b^*, Matteo Anderle^b,c,d,e^, Federico Capelli^b^, Alessandro Forti^b^, Chiara Bettega^b^, Corrado Alessandrini^d^, Maria del Mar Delgado^f^, Luca Pedrotti^g^, Piergiovanni Partel^h^, Giuseppe Bogliani^i^, Paolo Pedrini^b^, Mattia Brambilla^d,j^

*^a^ Department of Life Sciences, University of Trieste, Via L. Giorgieri 10, I-34127 Trieste, Italy.*

*^b^ Museo delle Scienze di Trento (MUSE), Ufficio Ricerca e collezioni, Corso del Lavoro e della Scienza 3, I-38122 Trento, Italy*

*^c^ Institute for Alpine Environment, Eurac Research, Drususallee/Viale Druso 1, I-39100, Bolzano/Bozen, Italy.*

*^d^ Milan University, Department of Environmental Science and Policy, Via Celoria 26, I-20123, Milan, Italy.*

*^e^ Department of Ecology, University of Innsbruck, Sternwartestrasse 15/Technikerstrasse 25, 6020, Innsbruck, Austria.*

*^f^ Biodiversity Research Institute (IMIB; CSIC-Oviedo University, Principality of Asturias), Campus Mieres, Mieres (Asturias), Spain.*

*^g^ Stelvio National Park, Via De Simoni 42, I-23032 Bormio, Italy.*

*^h^ Ente Parco Naturale Paneveggio-Pale di San Martino,* *località Castelpietra 2, I-38054, Primiero San Martino di Castrozza (Trento), Italy.*

*^i^ Department of Earth and Environmental Sciences, University of Pavia, Via Ferrata 1, I-27100 Pavia, Italy.*

*^j^ CRC Ge.S.Di.Mont., Milan University, Sede di Edolo, Via Morino 8, I-25048, Edolo (BS), Italy.*

***Corresponding author**: Davide Scridel ORCID: 0000-0003-3849-1178, email: [dscridel@gmail.com](mailto:dscridel@gmail.com)

**Table S1.** Breeding pairs according to site and relative habitat availability surrounding each nest (visually estimated in the field).

| **Breeding pair** | **Site** | **Visit** | **Bare %** | **Snow-bare %** | **Snow-grass %** | **Grass %** | **Snow %** | **Other %** | **Total** |
| --- | --- | --- | --- | --- | --- | --- | --- | --- | --- |
| PGA01 | Passo Gavia | a | 50 | 6.5 | 3.5 | 22 | 8 | 10 | 100 |
| PGA01 | Passo Gavia | b | 52 | 4.5 | 2.5 | 28 | 3 | 10 | 100 |
| PPO01 | Sasso Pordoi | a | 15 | 2.5 | 7.5 | 65 | 5 | 5 | 100 |
| PPO01 | Sasso Pordoi | b | 16 | 2 | 5 | 70 | 2 | 5 | 100 |
| PPO02 | Sasso Pordoi | a | 17 | 2.5 | 7.5 | 63 | 5 | 5 | 100 |
| PPO02 | Sasso Pordoi | b | 19 | 2 | 6 | 65 | 3 | 5 | 100 |
| PSE01 | Passo Sella | a | 15 | 2 | 7 | 62 | 3 | 11 | 100 |
| PSE01 | Passo Sella | b | 16 | 1 | 4 | 66 | 2 | 11 | 100 |
| PSE02 | Passo Sella | a | 0 | 0.5 | 7.5 | 70 | 2 | 20 | 100 |
| PSE02 | Passo Sella | b | 0 | 0.1 | 4.9 | 74 | 1 | 20 | 100 |
| PST03 | Passo Stelvio | a | 13 | 3 | 6 | 48 | 8 | 22 | 100 |
| PST04 | Passo Stelvio | a | 16 | 2 | 4 | 38 | 2 | 38 | 100 |
| PST06 | Passo Stelvio | a | 48 | 6 | 3 | 14 | 14 | 15 | 100 |
| PST06 | Passo Stelvio | b | 55 | 4 | 1 | 20 | 5 | 15 | 100 |
| PST07 | Passo Stelvio | a | 50 | 6 | 2.5 | 18.5 | 5 | 18 | 100 |
| PST08 | Passo Stelvio | a | 52 | 5.9 | 2.3 | 19.8 | 2 | 18 | 100 |
| PST10 | Passo Stelvio | a | 3 | 5 | 5 | 2 | 45 | 40 | 100 |
| PST10 | Passo Stelvio | b | 31 | 4.8 | 2.2 | 2 | 20 | 40 | 100 |
| PST11 | Passo Stelvio | a | 27 | 4.8 | 2.2 | 2 | 19 | 45 | 100 |
| PST12 | Passo Stelvio | a | 38 | 4.5 | 3 | 5 | 23.5 | 26 | 100 |
| PST13 | Passo Stelvio | a | 22 | 8 | 5 | 0 | 50 | 15 | 100 |
| PST13 | Passo Stelvio | b | 34 | 7 | 4 | 0 | 40 | 15 | 100 |
| ROS01 | Rosetta | a | 75 | 6 | 2 | 10 | 7 | 0 | 100 |
| ROS01 | Rosetta | b | 78.2 | 4 | 1.8 | 13 | 3 | 0 | 100 |
